# Clinical Guidelines for the Use of Antipruritic Drugs in the Control of the Most Frequent Pruritic Skin Diseases in Dogs

**DOI:** 10.3390/vetsci9040149

**Published:** 2022-03-22

**Authors:** Vincent Bruet, Marion Mosca, Amaury Briand, Patrick Bourdeau, Didier Pin, Noëlle Cochet-Faivre, Marie-Christine Cadiergues

**Affiliations:** 1 Veterinary Dermatology Referral Services, 44100 Nantes, France; vbruet@yahoo.fr; 2 Université de Lyon, VetAgro Sup, Interactions Cells Environment, UPSP 2016.A104, 69280 Marcy l’Etoile, France; marion.mosca@vetagro-sup.fr (M.M.); didier.pin@vetagro-sup.fr (D.P.); 3 Department of Dermatology, Ecole Nationale Vétérinaire d’Alfort, 94700 Maisons-Alfort, France; amaury.briand@vet-alfort.fr (A.B.); noelle.cochet-faivre@vet-alfort.fr (N.C.-F.); 4 Department of Clinical Sciences, ENVN (Oniris) Université de Nantes, 44307 Nantes, France; pjbourdeau44@gmail.com; 5 UMR BIPAR, Laboratoire de Santé Animale, 94700 Maisons-Alfort, France; 6 Department of Clinical Sciences, Université de Toulouse, ENVT, 31076 Toulouse, France; 7 INFINITy, Université de Toulouse, Inserm, CNRS, UPS, 31059 Toulouse, France

**Keywords:** dog, pruritus, allergy, atopic dermatitis, glucocorticoids, antihistamines, ciclosporin, oclacitinib, lokivetmab

## Abstract

Pruritus is a common clinical sign in many skin disorders and is currently the main complaint in canine dermatology. Pruritic skin diseases can affect the quality of life of dogs and their owners. Several families of antipruritic drugs are available to help control pruritus in dogs. The aim of this review is to help practitioners select the most appropriate symptomatic treatment in the most frequent situations of dermatological pruritus in dogs. The molecules reviewed here are systemic and topical glucocorticoids, antihistamines, ciclosporin, oclacitinib and lokivetmab. A level of evidence (1, 2 or 3) has been established according to a detailed algorithm for each individual study in the literature published between 1990 and March 2021. The guidelines result from evidence grading using the strength of recommendation taxonomy (SoRT) and clinical recommendations using a thorough methodology.

## 1. Introduction

Pruritus is defined as an unpleasant sensation that provokes the desire to scratch [1]. Pruritus is a common clinical sign of many skin disorders and is the most common complaint in canine dermatology. Pruritic skin diseases can affect the quality of life (QoL) of dogs and their owners [2]. The first indication for the use of antipruritic drugs is, of course, allergies, notably canine atopic dermatitis (AD). However, in pruritic infections or parasitic dermatoses, the speed of action and the level of efficacy of etiological treatments vary and, to improve the QoL of both the dog and the owner, the use of an additional symptomatic antipruritic drugs in addition to etiological treatment sometimes makes sense after obtaining a clear diagnosis.

The most common causes of pruritus are allergies, parasites and bacterial or fungal infections. Diagnosis requires a methodical workup, and successful treatment depends on the identification of the underlying causes. Antiparasitics, antibiotics and/or antifungals are the first step in the control of pruritus. The high prevalence of pruritus and its impact on animals should encourage veterinarians to carefully consider the appropriate symptomatic treatment. In 2007, in humans, members of the International Forum for the Study of Itch proposed an etiological classification of pruritus [3]. An adaptation of this classification for dogs was recently proposed, with six distinct origins: dermatological, systemic, neurological, psychogenic, mixed, and other [4]. The aim of this article is to guide the practitioner in the choice of the most appropriate symptomatic antipruritic in the most frequent situations of dermatological pruritus.

Antipruritic management is a multi-faceted approach that includes etiological, topical, and systemic symptomatic treatment. Even if topical treatments such as shampoos and emollients are helpful [5,6], this review only focuses on the main systemic and topical molecules with a veterinary drug registration and market authorization with antipruritic and/or antiallergic indications in Europe: glucocorticoids, antihistamines, ciclosporin, oclacitinib, and lokivetmab.

Different methods of synthesizing information and confronting conflicting opinions can be used [7]. Because of the absence or the paucity of literature with a high level of evidence, for this review, the seven experts used the consensus development conference leading to formalized consensus guidelines (not clinical practice guidelines) [7]. The experts conducted a critical analysis of the literature and formulated their recommendations. A minimum agreement among six out of the seven experts (>80%) was chosen to represent a consensus.

## 2. Materials and Methods

### 2.1. Consensus Method and Evidence Grading

Evidence in the literature was evaluated using the strength of recommendation taxonomy (SoRT) developed by editors of family medicine and primary care journals in the US [8]. The level of evidence (LoE) was graded using a three-point scale based on the quality of the methodology and the overall focus of the study as follows:

- Good-quality patient-oriented evidence;
- Limited-quality patient-oriented evidence;
- Other evidence, including consensus guidelines, opinion, case studies, or disease-oriented evidence.

The strength of recommendation (SoR) was ranked as follows [8]:

- Recommendation based on consistent and good-quality patient-oriented evidence;
- Recommendation based on inconsistent or limited-quality patient-oriented evidence;
- Recommendation based on consensus, opinion, case studies, or disease-oriented evidence.

### 2.2. Search Strategy

An extensive search was performed of the literature published between 1990 and October 2021 in PubMed, Scopus, and EBSCOhost Research Databases (Cab and Medline). Articles in English and French were searched using keywords. The names of the molecules or their chemical families were associated with combinations of the following search terms: dog, pruritus, allergy, atopic dermatitis, control, parasite, flea, sarcoptic mange, bacteria, *Malassezia*, vaccines, hepatotoxicity, renal failure, kidney, diabetes mellitus, mast cell tumor, histiocytoma, lymphoma, and urinary tract infection. Due to the paucity of data on antihistamines, research by the expert group was not limited to molecules available in veterinary and European drugs.

### 2.3. Inclusion Criteria

The following types of study were initially selected: randomized controlled trials, clinical trials, cohort studies, and case series studies. The authors elected to search the data from the original studies and to exclude meta-analyses unless a clear level of heterogeneity with a Q statistic test or an I^2^ index was established [9].

### 2.4. Exclusion Criteria

During the screening step, reviewers independently excluded studies for the following reasons: not a full-length article; duplicate study; article with no link with our topic, articles in a language other than English or French.

### 2.5. Clinical Situations

In addition to establishing a definitive diagnosis and an etiological treatment of the pruritus, the goal of this review was to establish the levels of evidence and the advice that can be given for the use of antipruritic drugs in:

- Flares in canine atopic dermatitis (AD);
- Long-term treatment of canine AD;
- Allergen-specific immunotherapy treatment (ASIT) of canine AD;
- Surface and superficial infectious skin disease (bacterial or *Malassezia* overgrowth, bacterial superficial pyoderma) with canine AD;
- Sensitization tests (e.g., intradermal skin test (IDST)) and elimination diet period;
- Parasitic skin diseases;
- Animals with neoplastic diseases (mast cell tumor, histiocytoma, and lymphoma) or chronic diseases (hepatic diseases, renal failure, urinary tract infection, and diabetes mellitus).

Deep infectious dermatoses are not addressed in this review. However, if a deep infectious dermatosis is already present or if one appears, the expert panel recommends that the infectious problem is treated before any antipruritic treatment is started.

### 2.6. Presentation of the Results

For each molecule and for each of the two sections “level of evidence” and “recommendations”, the following presentation scheme was selected:

- In atopic dogs, excluding the induction period of ASIT:
  ◦ Speed of action and efficacy;
  ◦ Reduction in the dose/frequency of administration;
  ◦ Combination of antipruritic molecules;
  ◦ Use in superficial bacterial pyoderma or microbial overgrowth;
  ◦ Adverse reactions.
- In atopic dogs, during the induction period of ASIT.
- In atopic dogs, during allergologic tests.
- In atopic dogs, during dietary trials.
- In pruritic ectoparasitic dermatoses.
- With vaccines.

## 3. Results: Levels of Evidence

### 3.1. Systemic Glucocorticoids

Thirty-three studies were reviewed. Data were only available for dexamethasone, methylprednisolone, and prednisolone. Table 1 reviews the retrieved articles according to the LoE in each individual study.

#### 3.1.1. In Atopic Dogs, excluding the Induction Period of ASIT

##### Speed of Action and Efficacy

Glucocorticoids were shown to be effective in the treatment of pruritus as early as four hours after oral administration in 25% of animals (LoE1 [23], LoE2 [14]) and within 3–7 days in 90% of animals (LoE1 [22], LoE2 [28,34]) at initial doses of 0.5–1 mg/kg, once (sid) or twice (bid) daily for 3–7 days for prednisolone, and 0.4–0.8 mg/kg sid for 5–7 days for methylprednisolone. A single intramuscular injection of dexamethasone at a dose of 0.2 mg/kg produced an effect on pruritus after one hour (LoE2 [10]). In several studies, the first monitoring point was later (≥2 weeks), a time frame that did not allow assessment of the speed of action [17,19,20,21,26,27].

At average initial doses of 0.5–1 mg/kg sid or bid for 3–7 days for prednisolone, 0.4–0.8 mg/kg sid for 5–7 days for methylprednisolone, and intramuscularly as a single injection at a dose of 0.2 mg/kg, the reduction in pruritus intensity and clinical score varied between studies and their observation criteria. On average, these doses resulted in a 50% reduction in lesions in 50% (LoE2) to 80% (LoE1) of dogs and in a 50% reduction in pruritus in 42% (LoE2) to 70% (LoE1) of dogs [10,14,15,16,17,20,21,22,23,24,25,26,27,28,29,30,31,32,33,34,35].

##### Reduction in the Dose/Frequency of Administration

After an induction dose lasting 3–7 days, depending on the study, the frequency of administration was gradually reduced according to either a pre-established schedule [14,15,16,17,19,20,21,22,23,25,28,29,30,31,34] or the clinical progression over a period of up to 16 weeks (LoE1 [11,12]). Most often, the reduction consisted of spacing out the doses to an every other day (eod) regimen (LoE1 [21,22,23,24,25,26]).

##### Combination of Antipruritic Molecules

Administration of prednisolone 1 mg/kg sid for seven days and then eod for the next 14 days, and ciclosporin 5 mg/kg sid for 28 days, resulted in better control of pruritus after two weeks (72.8%—23 dogs) than ciclosporin alone at the same dose (24.7%—25 dogs) (LoE2) [20].

Supplementation with a combination of borage and fish oils in 30 dogs receiving oral prednisolone significantly reduced pruritus and lesion scores by day 64 of supplementation, compared with a group of 30 dogs receiving prednisolone alone, and reduced the doses of prednisolone required over 12 weeks, although the difference was not statistically significant (LoE1) [35]. In an open-label study of 11 dogs receiving oral prednisolone (0.22–0.58 mg/kg at 48 h intervals) for several weeks or months, the addition of essential fatty acids (fish oil and evening primrose oil) resulted in a 24–100% reduction in the dose of prednisolone in eight out of 11 dogs after 12 weeks of supplementation (LoE2) [27]. The addition of a supplement containing zinc methionine, fatty acids and biotin to 11 atopic dogs receiving background treatment with oral glucocorticoids resulted in a reduction in clinical and pruritus scores in 6 out of 11 dogs (55%) after 12 weeks of glucocorticoid dose reduction (LoE2) [33].

##### Use in Superficial Bacterial Pyoderma or Microbial Overgrowth

There are no published studies on the subject.

##### Adverse Reactions

Where specified, adverse events were reported in between 10% (LoE2) [18] and 100% (LoE1) [11,24] of treated dogs. Polyphagia and polyuria–polydipsia syndrome were among the most common (LoE1) [22,24,26], (LoE2) [15]. Other reported adverse events were digestive disorders (vomiting, diarrhea, loose stools) (LoE1) [22,23,26], superficial pyoderma in 10 (LoE1) [23] to 41% (LoE2) [33] of cases. A summary is given in Table 2.

#### 3.1.2. In Atopic Dogs, during the Induction Period of ASIT

No data were available to assess the efficacy of systemic glucocorticoids in atopic dogs during the induction period of ASIT. However, oral prednisolone at a dose of 1 mg/kg was effective in controlling the adverse effects of rush immunotherapy in eight dogs (LoE2) [37]. The dose of methylprednisolone given in months 5 and 6 was reduced by 58% compared with the amount given during the first two months (LoE2) [36]. Furthermore, in the long term, a reduction in the dose of prednisolone from 1–2 mg/kg/week to 0.4 mg/kg/week was possible (LoE2) for 22 dogs out of 117 dogs undergoing ASIT that were monitored for 48 months [38].

#### 3.1.3. In Atopic Dogs, during Allergologic Tests

Administration of prednisolone to 15 dogs at 1.1 mg/kg for seven days then eod for 14 days had no influence on the results of serological tests (allergen-specific IgE) performed at day 21 compared with the results obtained at day 0 (LoE2) [39]. There were highly significant effects of prednisolone at a dose of 1 mg/kg in inhibiting the skin reactivity against histamine and *Dermatophagoides farinae* after one week, as compared with placebo. Skin reactions increased again on the 14th day, although there was still a statistically significant difference to baseline. Prednisolone should be discontinued at least two weeks prior to IDST (LoE2) [40].

#### 3.1.4. In Atopic Dogs, during Dietary Trials

Prednisolone, at a dose of 0.5 mg/kg sid or bid for three days, followed by 0.5 mg/kg daily for 5–17 days, then eod for 6–8 days, reduced the duration of the exclusion diet regime to 4–6 weeks instead of the usual eight weeks (LoE2) [41,42].

#### 3.1.5. In Pruritic Ectoparasitic Dermatoses

No published data are available for the antipruritic use of systemic glucocorticoids in dogs with ectoparasites.

#### 3.1.6. With Vaccines

No published data are available for the effect of the antipruritic use of systemic glucocorticoids on vaccinations.

### 3.2. Topical Glucocorticoids

Nine studies were included. Data were mainly available for hydrocortisone aceponate. Table 3 summarizes the articles reviewed according to the LoE of each individual study.

#### 3.2.1. In Atopic Dogs, excluding the Induction Period of ASIT

##### Speed of Action and Efficacy

The efficacy of hydrocortisone aceponate on pruritus and skin inflammation at 14 days compared with the final efficacy at 28 days was 27% and 82%, respectively (LoE1) [45].

Hydrocortisone aceponate 0.0584% solution, applied daily on skin lesions, at an average dose of two applications per 100 cm^2^, significantly reduced pruritus and the Canine Atopic Dermatitis Extent Severity Index (CADESI) score after 14 days of treatment. CADESI was reduced by more than 50% in 53.3% (LoE1) [45] to 71.4% (LoE2) [46] of the dogs, and pruritus score improvement ≥50% was obtained in 33.3% (LoE1) [45] to 76.2% (LoE2) [46] of dogs. After 28 days, a ≥50% improvement in the clinical and pruritus score was observed in 58.3% (LoE2) to 73.3% (LoE1) and 33.3% (LoE2) to 46.7% (LoE1) of the treated dogs, respectively (LoE1) [45], (LoE2) [47]. The efficacy remained stable from day 28 to day 84 with decreasing doses (see below—reduction in the dose): a ≥50% improvement in the clinical and pruritus score was observed in 75% and 65.2% of the treated dogs, respectively (LoE2) [47]. A daily application until remission of skin lesions followed by biweekly applications of hydrocortisone aceponate increased the time to relapse to 115 days (median) versus 33 days (median) in the untreated group (LoE1) [43]. Topical treatment with a 0.015% triamcinolone acetonide solution applied on lesional skin for 28 days was effective. Clinical improvement was observed in 67.3% of treated dogs versus 23.5% in the placebo group (LoE1) [49].

##### Reduction in the Dose/Frequency of Administration

The frequency of administration of hydrocortisone aceponate could be progressively decreased without loss of efficacy in the majority of treated dogs: after 28 days with an eod application in 38% of dogs, and after 56 days with an eod to biweekly application in 54% (LoE2) [47]. After 70 days of treatment, only 14% of dogs needed a daily application, 33.3% needed an eod treatment, and 33.3% a twice weekly application (LoE1) [45].

Another study showed that an initial treatment period with a daily application of hydrocortisone aceponate enabled maximum efficiency: after 21 days of one application daily, CADESI was reduced by 29.2% compared with 14.8% when an intermittent regimen (three days on/four days off) was used (LoE2) [48]. In the study using 0.015% triamcinolone acetonide solution, the results were obtained with a decrease in the frequency of application by stages bid for seven days, then sid for seven days, and finally eod for two weeks [49] (LoE1).

##### Combination of Antipruritic Molecules

In association with oclacitinib (see Section 3.5.1), applications of hydrocortisone aceponate sid for seven days then eod for 21 days prevented the rebound effect observed when oclacitinib administration frequency was reduced from bid to sid (LoE1) [44]. After 28 days, a 54.1% and 56% reduction in pruritus and CADESI scores, respectively, was observed in the aceponate hydrocortisone + oclacitinib group versus 30.8% and 30.5% in the oclacitinib group (LoE1) [44].

##### Use in Superficial Bacterial Pyoderma or Microbial Overgrowth

There are no published studies on the subject.

##### Adverse Reactions 

Short-term, self-resolving adverse events (diarrhea, pyrexia, vomiting and persistent estrus) were reported with hydrocortisone aceponate (Table 2) but were thought not to be related to treatment (LoE1) [45]. Skin atrophy (clinical and histopathological examination) was reported after 14 days of treatment (LoE3) [50]. One study reported that post ACTH stimulation, the cortisol level decreased (by 36%) after three weeks of daily application of hydrocortisone aceponate, but not if the frequency of application was reduced (LoE2) [48].

With triamcinolone, adverse reactions were reported in 5/52 dogs after 28 days of treatment despite progressive decreasing frequencies of application on lesional skin: squamosis (two dogs), polyphagia and polyuria–polydipsia syndrome (two dogs), and digestive troubles (one dog) (LoE1) [49].

#### 3.2.2. In Atopic Dogs, during the Induction Period of ASIT

There are no published studies on the subject.

#### 3.2.3. In Atopic Dogs, during Allergic Testing

After seven days of daily applications of hydrocortisone aceponate on the skin of a colony of ten laboratory atopic dogs, skin reactivity decreased after skin injection of proinflammatory molecules (histamine, anticanine-IgE polyclonal antibodies) with a return to normal reactivity after a wash-out period of 14 days (LoE3) [50].

#### 3.2.4. In Atopic Dogs, during Dietary Trials

There are no published studies on the subject.

#### 3.2.5. In Pruritic Ectoparasitic Dermatoses

Hydrocortisone aceponate rapidly relieved pruritus and skin lesions in experimentally induced flea bite hypersensitivity (FBH) in dogs treated with nitempyram. Pruritus was reduced by 94% in the treatment group versus 24% in the control group in terms of cumulative time, and by 86% versus 34% in terms of frequency. After three days, the clinical score was reduced by 23% in the treatment group versus 0% in the control group and by 43% versus 15% after seven days (LoE2) [51].

#### 3.2.6. With Vaccines

There are no published studies on the subject.

### 3.3. Antihistamines

Twenty-seven studies were included. Table 4 summarizes the retrieved articles according to the level of evidence of each individual study.

#### 3.3.1. In Atopic Dogs, excluding the Induction Phase of ASIT

##### Speed of Action and Efficacy

The majority of studies were conducted over short periods (10 to 14 days) and reported the effectiveness of the molecules, tending to suggest the antihistamines acted rapidly (LoE1) [63], (LoE2) [57]. However, some studies reported that the effect was only observed or was greater over longer periods (three to six weeks) (LoE2) [17,64].

With hydroxyzine (±2 mg/kg bid or tid, 0.9–5 mg/kg), the molecule metabolized in cetirizine, considered to be the active metabolite, a more than 50% improvement was observed in 28–80% of cases (LoE2) [57,61]) and the absence of an effect was reported in one article (LoE3) [56]. Cetirizine, 1 to 3 mg/kg/day, was poorly effective (18%) for the management of pruritus (>50% of improvement) (LoE2) [55] or no better than the placebo (LoE1) [53], (LoE3) [56].

A more than 50% improvement in pruritus was noted in 16–80% of dogs with chlorpheniramine (±0.2 mg/kg bid or tid, 0.14 mg to 1.6 mg/kg) (LoE2) [57,61], in more than 80% of dogs with promethazine (±1 mg/kg tid, 0.58 mg/kg to 10.4 mg/kg) (LoE2) [57], in 30% of dogs with diphenhydramine (±2 mg/kg tid) (LoE2) [61] and in 38% of dogs with AHR-13268 (LoE2) [52].

Data on cyproheptadine (0.2 mg/kg sid to tid, 0.1 to 2.1 mg/kg), trimeprazine (0.27 to 4.1 mg/kg tid) and terfenadine (5 mg/kg bid) diverged, showing it to be efficient in a large number of cases (>80%) (LoE2, LoE3) [57,66,67,68] or inefficient (LoE2) [18,62,65].

Pheniramine (2 mg/kg/day) was inefficient (LoE3) [34].

With fexofenadine (18 mg/kg/day), the pruritus was reduced by half after six weeks (LoE2) [17,19].

Oxatomide (2 to 3 mg/kg/day) was reported to be effective for pruritus in 33% of allergic dogs in an open trial (LoE2) [64].

The pruritus score was reduced by around 30% with clemastine (0.5 to 1.5 mg/dog) in four studies (LoE2) [18,57,59,61]) and 72% in one study (LoE2) [57].

With dimetindene (1 mg/10 kg), there was a 38% clinical improvement and a 22% pruritus improvement in 66% of the dogs (statistical difference from the placebo group) (LoE1) [63]. In contrast, there was no difference between the treated group and the placebo in the rate of animals improved by more than 50% (LoE1) [63]. The benefit seemed to vary with the dog and was considered to be animal-dependent, and hence not predictable (LoE1) [63].

##### Reduction in the Dose/Frequency of Administration

None of the listed studies tested a reduction in the dose or frequency of antihistamine administration.

##### Association of Antipruritic Molecules

The combination hydroxyzine + chlorpheniramine (0.14 mg chlorpheniramine and 4.2 mg hydroxyzine per kg) enabled the improvement of the clinical signs by between 18% (LoE2) [69] and 47% (LoE1) [63], and of pruritus by 24.7% (LoE2) [69] or 30% (LoE1) [63]. A reduction of more than 25% in pruritus was observed in 59% (statistical difference between the treated and placebo group) (LoE1) [63]. In contrast, no difference from the placebo group was found in the rate of animals showing more than 50% improvement (LoE1) [63]. The benefit was described as animal-dependent and not predictable (LoE1) [63].

With the use of glucocorticoids, a slight improvement in the management of pruritus was observed (LoE2) [18,54,57], sometimes with a lower dose of glucocorticoids (30%) (LoE2) [18].

Therapy combined with essential fatty acids was also studied and showed that a possible synergism further improved the clinical signs and management of pruritus (LoE2) [58,60].

##### Use in Superficial Bacterial Pyoderma or Microbial Overgrowth

One article reported the lack of impact of infections on the efficacy of antihistamines (LoE2) [61]. However, no data are available on the impact of antihistamines on the management of infections.

##### Adverse Reactions 

Sedation was the main but infrequently reported side effect (LoE1) [53,63] (Table 2). This effect was observed more frequently with the first generation of antihistamines and could be modulated by reducing the dose, sometimes without affecting the effectiveness of the molecule (LoE1) [63].

#### 3.3.2. In Atopic Dogs, during the Induction Period of ASIT

One article reported the absence of association between the response to antihistamines and the response to ASIT (LoE2) [61].

#### 3.3.3. In Atopic Dogs, during Allergologic Tests

Inhibition of IDST reactions was observed with the administration of cetirizine, hydroxyzine, loratadine, and terbinafine (LoE3) [40,70,71,72,73]. This effect ended between three and ten days depending on the molecule and the dose (LoE3) [40,70,71,72,73]. There are no studies on the impact of antihistamines on serological tests.

#### 3.3.4. In Atopic Dogs, during Dietary Trials

There are no published studies on the subject.

#### 3.3.5. In Pruritic Ectoparasitic Dermatoses

Pruritus caused by some cases of flea bite hypersensitivity and sarcoptic mange seemed to be managed by terfenadine in seven to ten days (LoE3) [66,67,68].

#### 3.3.6. With Vaccines

There are no published studies on the subject.

### 3.4. Ciclosporin A (Aka Cyclosporin A, Cyclosporin, Ciclosporin)

Thirty-one studies were included. Table 5 summarizes the retrieved articles according to the level of evidence of each individual study.

#### 3.4.1. In Atopic Dogs, excluding the Induction Period of ASIT

##### Speed of Action and Efficacy

The efficacy of ciclosporin was rapid, with, relative to the final efficacy, 35–45% from D7 (depending on the study), 45–60% at D15 and 65–95% at D28 for the pruritus score (LoE1) [12,20,47,74,75,81,85,89] and 50–75% at D7, 70–85% at D28 for the clinical score (LoE1) [12,20,47,74,75,81,85,89,94]. The average final reduction in pruritus varied from 42% (LoE1) to 78% (LoE1)/100% (LoE2) [20,26,74,75,77,82,83,85,89] and that of the clinical score from 44% (LoE2)/56.5% (LoE1) to 74.3% (LoE1)/83% (LoE2) [20,26,33,74,75,77,81,83,85,88,89,94]. The score was halved in 40% (LoE1) to 76.9% (LoE1)/93.7% (LoE2) [93] of the dogs for pruritus [12,24,47,74,77,81] and 45% (LoE1) to 84.6% (LoE1)/87.5% (LoE2) for the lesions [12,24,26,47,74,77,78,89,93]. One study suggested that only dogs with an intrinsic (non-IgE) (but not extrinsic) form improved when given ciclosporin (LoE2) [84].

##### Reduction in the Dose/Frequency of Administration

Over time, it was possible to reduce the dose (LoE1) [12,47,81,84]. By the second month of treatment, approximately 50% of dogs were controlled with an eod dose (LoE1) [12,47]. At three months and beyond, treatment remained daily for 32–35% of dogs, eod for 35–42% and biweekly for 24–29% (LoE1) [12,81,84]. The average daily dose was reduced from 5 mg/kg/day to 3.5–4 mg/kg/day after two months (LoE1) [76,82]. In one study, 61.8% of the dogs relapsed after an average of 40.7 days off ciclosporin [13].

##### Association of Antipruritic Molecules

A three-month EFA supplementation (ratio 3.75/1, omega-3/6), in dogs already receiving ciclosporin for two months, made it possible to reduce ciclosporin by 0.85 mg/kg/day (LoE1) [76]. As previously stated (Section 3.1.1), the initial combination of ciclosporin (5 mg/kg/day) and prednisolone at 1 mg/kg/day, then eod for 14 days, accelerated clinical improvement (LoE1), the only observed effect being lethargy (17.4%) [20]. After seven days, the combination reduced pruritus by 64.4% compared with 14.5% with ciclosporin alone [20]. In healthy dogs, the combination of ciclosporin (5 mg/kg/day) and oclacitinib over three weeks did not alter any clinical or biochemical side effects compared with oclacitinib used alone (LoE2) [86].

##### Use in Superficial Bacterial Pyoderma or Microbial Overgrowth

There have been no reports of reduced or abnormal responses to antibiotic, antifungal, local antiseptic, or systemic therapy with ciclosporin in clinical studies (LoE2) [12,20,33,74,75,78,80]. To our knowledge, responses to anti-infectious agents used alone or with ciclosporin or another antipruritic drug have never been compared.

##### Adverse Reactions 

Common digestive side effects may appear within one month (30–50%) (LoE1) (Table 2). The most common signs are vomiting (30–40% of dogs), and diarrhea (20–35%) [12,20,24,26,31,33,47,74,75,76,78,80,82,84,85,87,92,93,97,98]. They are mostly episodic (70% less than three vomiting episodes) and moderate [78]. Anorexia/dysorexia, polyphagia, flatulence, hypersalivation, abdominal pain and weight loss are also mentioned [12,20,24,26,31,33,47,74,75,76,78,80,82,84,85,87,92,93,97,98]. Infectious skin complications (bacterial, fungal) are not rare (0% to 29% of cases) [12,20,24,26,33,47,74,75,76,78,80,84,87,98]. The bacteriuria sometimes observed did not differ from that observed in the control group in one study (LoE1) and clinical signs may exceptionally be associated (LoE1) [78,87,98]. While remaining rare (about 1%) (LoE1), gingival hyperplasia or papillomatous lesions (LoE1) are the most frequently described short- and medium-term neoformations [12,20,26,75,76,78,87,99]. They regressed, at least partially, when ciclosporin was stopped [12,20,33,74,75,78,80]. Medium- to long-term hypertrichosis (LoE1) was rarely reported, but may not have been recognized in early studies [76,87]. Other very rarely mentioned effects were lethargy, weakness, skin lesions, pruritus and neurological problems.

#### 3.4.2. In Atopic Dogs, during the Induction Period of ASIT

There is no clinical evidence of an impact of ciclosporin on the efficacy of desensitization.

#### 3.4.3. In Atopic Dogs, during Allergologic Tests

According to the only available study, IDST or Immunoglobulin E assays are not modified by ciclosporin (LoE1) [96].

#### 3.4.4. In Atopic Dogs, during Dietary Trials

There are no published studies on the subject.

#### 3.4.5. In Pruritic Ectoparasitic Dermatoses

There are no published studies on the subject.

#### 3.4.6. With Vaccines

There are no published studies on the subject.

### 3.5. Oclacitinib

Seventeen studies were included. Table 6 summarizes the retrieved articles according to the level of evidence of each individual study.

#### 3.5.1. In Atopic Dogs, excluding the Induction Period of ASIT

##### Speed of Action and Efficacy

The recommended dose is 0.4–0.6 mg/kg bid for 14 days then 0.4–0.6 mg/kg sid.

The reduction in pruritus, assessed using the pruritus Visual Analog Scale (pVAS), was very rapid, with a reduction observed as early as four hours post-administration (LoE1) [23,74,100,101,102,103]. The reduction then slowed downed markedly until day 14, increased slightly at day 28, and pruritus then continued to decrease moderately (LoE1) [74,101]. The longest study showed a 57.8% reduction in pruritus at day 630 (LoE2) [102].

In the case of clinical lesions, efficacy was assessed with CADESI-02 and showed a reduction of 48.4% (LoE1) [101] to 58.7% (LoE1) [74] at day 14, 48.4% (LoE1) [101] to 58.3% (LoE1) [74] at day 28, 63.8% at day 56 (LoE1) [74] and 67% at day 84 (LoE1) [74]. On day 112, a 60% improvement was reported (LoE1) [101].

Oclacitinib enabled improvement in clinical lesions. Improvement was intense until day 14, after which clinical scores continued to decrease less markedly (LoE1). The percentages of dogs that achieved a reduction of ≥50% from baseline in CADESI-02 were 71% on day 14, 65% on day 28, 77% on day 56 and 82% on day 84 (LoE1) [74]. On day 630, a 68.5% improvement in clinical lesions was observed (LoE2) [102].

Some experimental models of canine AD were developed to study oclacitinib efficacy and showed that oclacitinib could delay the development of clinical lesions after allergen contact (LoE2) [105] and increase hydration (LoE2) [32], but no conclusive results were found concerning transepidermal water loss (TEWL) evaluation (LoE2) [32,105].

QoL was assessed in recent studies and demonstrated that more than 91% of owners were confident that treatment with oclacitinib was effective and improved QoL (LoE2) [102].

##### Reduction in the Dose/Frequency of Administration

A moderate exacerbation of pruritus [101,103] and a slight increase in or stabilization of clinical lesions scores [74,101] were observed when tapering oclacitinib therapy bid in dogs after day 14 to sid.

##### Association of Antipruritic Molecules

Hydrocortisone aceponate spray applied at the same time as oclacitinib prevented the relapse of pruritus and clinical lesions after tapering oclacitinib (mean reduction from the baseline of the hydrocortisone aceponate spray and oclacitinib-treated group was significantly higher than that observed in the oclacitinib and placebo spray-treated group for the PVAS and CADESI-4 on D21 (59.9% versus 27.6%, *p* = 0.0216) and D28 (56.0% versus 30.5%, *p* = 0.0109), respectively) (LoE2) [44].

Supplementation with SF68 *Enterococcus faecium* was associated with no difference in oclacitinib dose reduction versus placebo in 21 client-owned dogs with canine AD. Clinical disease scores did not differ between the groups upon completion of the study. There was no significant change in PVAS from the baseline in either group at any time point (LoE2) [108].

Treatment with oclacitinib alone or concomitantly with ciclosporin was not associated with adverse events, except diarrhea in two healthy dogs receiving both treatments (LoE2) [86]. Oclacitinib therapy combined with hydrocortisone aceponate spray was not associated with adverse events (LoE2) [44].

##### Use in Superficial Bacterial Pyoderma or Microbial Overgrowth

No available studies evaluated the impact of the use of oclacitinib on infectious dermatosis. However, one study compared the use of antibiotics in allergic dogs treated with oclacitinib versus allergic dogs treated with another antipruritic drug and reported that the use of antibiotics was reduced in dogs with allergic skin disease treated with oclacitinib compared with treatment with other antipruritic drugs (LoE2) [106].

##### Adverse Reactions 

Reported adverse effects were dermatological signs (LoE1, LoE2) (otitis [101,103], pyoderma [101,103], pododermatitis [101]), gastrointestinal signs (LoE1, LoE2) (anorexia, vomiting [100,101,102,103] and diarrhea [100,101,102]), which were mild or moderate in severity and resolved in one day without treatment (LoE1) [101], and urinary tract infection/cystitis (LoE1, LoE2) [100,102] (Table 2). One study proved that bacteriuria was not an expected adverse effect in dogs without a prior history of urinary tract infection or predisposed condition (LoE2) [107]. There was no association between the daily administration of oclacitinib and the odds of malignancies or benign skin masses (LoE2) [104]. Blood analysis (complete blood count or biochemistry) of dogs treated with oclacitinib showed values in reference ranges (LoE1, LoE2) [23,103].

#### 3.5.2. In Atopic Dogs, during the Induction Period of ASIT

Oclacitinib was used in 33 dogs during subcutaneous immunotherapy (fast-escalation or conventional regimen). No specific information is available to determine if oclacitinib inhibits or reduces the severity of adverse events in subcutaneous-immunotherapy-treated dogs (LoE2) [19].

#### 3.5.3. In Atopic Dogs, during Allergologic Tests

In experimental canine models of AD epicutaneously sensitized with house dust mites, *Dermatophagoides farinae*, no statistically significant difference in allergen-specific IgE was found between treated (oclacitinib) and placebo groups and over time (LoE2) [105].

#### 3.5.4. In Atopic Dogs, during Dietary Trials

Oclacitinib was able to shorten the elimination diet trial time with a provocation starting after four weeks and diagnosis within six weeks in most cases (LoE2) [42].

#### 3.5.5. In Pruritic Ectoparasitic Dermatoses

Oclacitinib, associated with systemic antiparasitic drugs, allowed a significant decrease in pruritus within 24 h, provided rapid relief from pruritus, and reduced inflammation in dogs with sarcoptic mange (LoE2) [110]. In other ectoparasitic diseases, no studies have been conducted to assess the progression of clinical signs with oclacitinib.

#### 3.5.6. With Vaccines

There are no published studies on the subject.

### 3.6. Lokivetmab

Nine studies were included. Table 7 summarizes the retrieved articles according to the level of evidence of each individual study.

#### 3.6.1. In Atopic Dogs, excluding the Induction Period of ASIT

##### Speed of Action and Efficacy

Lokivetmab is administered subcutaneously at 28-day intervals at a dose of 1 or 2 mg/kg depending on the country.

Lokivetmab was effective on pruritus (LoE1) [112] with a ≥50% score reduction in owner pruritus VAS score for 57% of 2 mg/kg treated dogs. The reduction in pruritus score was noted 24 h after a subcutaneous injection at a mean dose of 2.2 (1.8–3.7) mg/kg in 56% of dogs (LoE3) [116], within one to three days in 39.7% of dogs, and after three days in 100% of dogs (LoE3) [116]. Lokivetmab, at a dose of 2 mg/kg, reduced the lesional score by 37% and 43%, seven and fourteen days after the injection, respectively (LoE1) [112].

The level and duration of the response are dose-dependent. The global efficacy of one lokivetmab injection, at a dose of 1 to 3.3 mg/kg by the owner and investigator, were 67.8% and 70.1%, respectively (LoE1) [113].

A reduction in pruritus was observed in 87.8% of the dogs after one injection at a mean dose of 2.2 (1.8–3.7) mg/kg (LoE1) [75] with a pVAS score reduction of ≥50% in 77% of the dogs (LoE1) [75].

Lokivetmab improved the skin condition of atopic dogs (LoE1) [75,112], (LoE2) [114,115]. The level and duration of the response increased with increasing dose. A lesional score reduction of ≥50% was observed in 46% and 57.3% of the dogs 28 days after an injection, at a dose of 2 mg/kg (LoE1) [112] and 1.32 mg/kg (1–2.2 mg/kg) (LoE2) [114], respectively. A ≥50% reduction in the lesional score was observed in 78.8% and 80.8% of the dogs after two and three monthly injections, respectively, at a dose of 1.32 mg/kg (1–2.2 mg/kg) (LoE2) [114]. The skin condition was considered normal in 36.6% of the dogs after two injections at an interval of one month, at a dose of 2 mg/kg (LoE3) [116], and in 59.3% of the dogs after eight injections (LoE3) [116].

A proactive monthly treatment with lokivetmab prevented the development of clinical signs in 43%, 33%, 28%, and 28% of the dogs, at three months, six months, nine months, and 12 months, respectively (LoE2) [115].

##### Reduction in the Dose/Frequency of Administration

There are no published studies on the subject.

##### Association of Antipruritic Molecules

There are no published studies on the subject.

##### Use in Superficial Bacterial Pyoderma or Microbial Overgrowth

There are no published studies on the subject.

##### Adverse Reactions 

Reported adverse reactions varied with the study (Table 2). They were similar in frequency and severity between lokivetmab- and placebo-treated groups (LoE1) [111,112,113], (LoE3) [117]. The most frequently cited adverse events were otitis externa, dermatitis, pyoderma, erythema, vomiting, anorexia, lethargy, pruritus, and diarrhea. The adverse events were classified as “mild” or “moderate” and resolved spontaneously in 87% of cases, with the remaining cases responsive to supportive care (LoE1) [111]. All hematology, serum biochemistry, and urine analysis parameters remained within the normal reference range (LoE1) [111,112,116].

#### 3.6.2. In Atopic Dogs, during the Induction Period of ASIT

Lokivetmab seemed to have no effect on the 15 dogs on ASIT, in neither fast-escalation subcutaneous immunotherapy nor a conventional subcutaneous immunotherapy regimen (LoE3) [109].

#### 3.6.3. In Atopic Dogs, during Allergologic Tests

There are no published studies on the subject.

#### 3.6.4. In Atopic Dogs, during Dietary Trials

There are no published studies on the subject.

#### 3.6.5. In Pruritic Ectoparasitic Dermatoses

There are no published studies on the subject.

#### 3.6.6. With Vaccines

There are no published studies on the subject.

## 4. Recommendations

The results are summarized in Table 8 and Table 9.

### 4.1. Systemic Glucocorticoids

#### 4.1.1. In Atopic Dogs, excluding the Induction Period of ASIT

##### During Flares (Reactive Therapy)

Oral glucocorticoids (prednisolone, methylprednisolone (SoR A), prednisone (SoR B) and injectable dexamethasone (SoR C) are effective for the treatment of flares of canine AD. They can be administered initially at 0.5–1.0 mg/kg (prednisolone) or 0.4–1 mg/kg (methylprednisolone) per day (SoR A), in one or divided into two administrations (SoR A), using a dose that is maintained or that can be decreased after 3–7 days (SoR B) using either a standard protocol or based on the outcome (SoR B). A significant reduction in pruritus is obtained rapidly, in a few hours or in a few days (SoR B).

##### In Non-Flare Periods (Proactive Therapy)

No data are available. Use of systemic glucocorticoids for long-term management of canine AD is not recommended (SoR C) or only with great care, if a low dose (SoR C) is administered at 48 or 72 h intervals. Adrenal insufficiency can be minimized by instituting therapy eod, but adrenal insufficiency may occur at any pharmacologically active dose. Adrenal insufficiency may be particularly obvious after the discontinuation of corticosteroid therapy. Dose reduction and discontinuation should be gradual to avoid precipitating adrenal insufficiency even temporarily (SoR C).

Adverse events are frequent in short-term treatments and mainly consist of polyuria, polydipsia, polyphagia (SoR A) and gastrointestinal disorders (SoR B).

##### In Dogs with Superficial Pyoderma or Microbial Overgrowth

Few data are available. Daily oral administration of 0.75 mg/kg prednisolone for one month did not alter the skin microbiome of six asymptomatic atopic dogs [118]. Nevertheless, considering their potential pro-infectious characteristics, their use should be restricted in the case of secondary skin infections and infectious diseases (SoR C).

##### In Dogs with Specific Problems 

Liver disorders

Changes in liver parameters associated with the use of systemic glucocorticoids are commonly observed. In the case of long-term use, their detection by regular liver function tests is recommended. The use of glucocorticoids is not recommended in animals with liver failure (SoR C).

Renal disorders

The use of glucocorticoids is not recommended in dogs with renal disorders (SoR C).

Diabetes mellitus

Glucocorticoids stimulate gluconeogenesis. Lipogenesis is enhanced in certain areas of the body and adipose tissue may be redistributed away from the extremities to the trunk (SoR C). The use of glucocorticoids is associated with an increase in serum insulin but not serum fructosamine concentrations in dogs with canine AD (SoR B). Since prolonged use of systemic glucocorticoids can lead to the development of diabetes, their use in a diabetic animal, even if controlled, is not recommended (SoR C).

Neoplastic diseases

Long-term use can cause immunomodulation that can lead to the development of neoplasia (SoR C). The use of glucocorticoids is not generally recommended in dogs with cancers (SoR C) unless they are part of the chemotherapy regimen [119,120].

Urinary infections

Routine urine cultures and assessment of bacteriuria by cystocentesis should be part of the monitoring for dogs on long-term glucocorticoids. Glucocorticoids should be used cautiously in dogs with occasional urinary tract infections and be avoided in dogs with recurrent urinary infections (more than two episodes in six months or more than three episodes per year) (SoR C) [121].

#### 4.1.2. In Atopic Dogs, during the Induction Period of ASIT

Systemic glucocorticoids can be used during ASIT (SoR B) and can be used in the case of an adverse event during ASIT rush therapy (SoR B).

#### 4.1.3. In Atopic Dogs, during Allergy Testing

Systemic glucocorticoids do not interfere with specific allergen serology tests (SoR B). They should be stopped at least two weeks prior to IDST as they can interfere with cutaneous reactions (SoR B).

#### 4.1.4. In Atopic Dogs, during Dietary Trials

Systemic glucocorticoids can be used during the initial period of an elimination diet (SoR C).

#### 4.1.5. In Pruritic Ectoparasitic Dermatoses

No data are available. Long-term use may indirectly promote parasitic infestation while masking or reducing symptoms. With the exception of demodicosis (confirmed or previous), for which glucocorticoids should be avoided, a short course (five days maximum) of oral glucocorticoids would be acceptable when initiating the treatment of a highly pruritic sarcoptic mange, flea infestation or flea allergy dermatitis (SoR C).

#### 4.1.6. With Vaccines

In the case of vaccination with live attenuated vaccines, an interval of two weeks should be observed before or after treatment (SoR C). In the guidelines for the vaccination of dogs and cats published in 2016, experts reported the suggestion that immunosuppressive glucocorticoid treatment, i.e., with higher doses than for allergic diseases, prior to or concurrently with vaccination, has no significant suppressive effect on antibody production in response to vaccines. However, revaccination is recommended two or more weeks after glucocorticoid therapy has ended, especially when treatment occurs during the administration of the initial series of core vaccines [122].

### 4.2. Topical Glucocorticoids

#### 4.2.1. In Atopic Dogs, excluding the Induction Phase of ASIT

##### During Flares (Reactive Therapy)

Topical glucocorticoids (hydrocortisone aceponate (SoR C), triamcinolone (SoR C)) are effective for the treatment of flares of canine AD, especially for localized lesions. At the time of writing (2022), the lack of data on the efficacy of topical glucocorticoids in the first two weeks of treatment does not allow a recommendation beyond SoR C. The optimal regimen is daily treatment followed by a reduction in the frequency of application on the skin based on outcome (SoR C).

##### In Non-Flare Periods (Proactive Therapy)

Hydrocortisone aceponate can be recommended in non-flare periods (SoR A). The frequency of application can be reduced after one or two weeks of treatment, which reduces the risk of adverse reactions (SoR B). Twice weekly applications on previous lesional skin areas reduce relapses (SoR A). Skin thinning can be observed after 14 days of treatment (SoR B).

The use of triamcinolone is also possible and effective, although there may be an increase in side effects (SoR B). With the use of other topical glucocorticoid formulations, there may be variations in efficacy and safety depending on the strength of the glucocorticoid, the chemical structure, and the vehicle [123,124]. Most topical corticosteroids are absorbed in quantities that can produce both systemic and local adverse effects [125]. These marked adverse effects may be observed with prolonged or frequent use and are more rapidly observed, more frequent, and more severe with potent topical corticoids (SoR C).

##### In Dogs with Superficial Pyoderma or Microbial Overgrowth

No data are available but, for the same reasons as systemic corticosteroids, it is not recommended to use topical glucocorticoids in the case of secondary skin infections and infectious diseases (SoR C).

##### In Dogs with Specific Problems

No data are available, but hydrocortisone aceponate seems to be a safe molecule because of its particular metabolic pathway, i.e., in the skin with no systemic absorption (SoR C). Because of its potential local effect on skin (thinning), its use is not recommended in dogs suffering from other systemic diseases that have an impact on skin integrity (e.g., Cushing disease) (SoR C). Like with the use of other topical glucocorticoids, caution is required because systemic absorption, potencies and the risk of adverse reaction will vary with the glucocorticoid molecule (SoR C).

Liver disorders

No data are available but as hydrocortisone aceponate does not cause changes in biochemical parameters, this molecule could be used in dogs with liver disorders (SoR C). Caution is required with other topical glucocorticoids (SoR C).

Renal disorders

No data are available. Hydrocortisone aceponate does not cause changes in renal parameters so this molecule could be used in dogs with renal disorders, but regular biochemical tests are mandatory (SoR C).

Diabetes mellitus

No data are available. Due to its mode of action, hydrocortisone aceponate could be used in dogs with diabetes mellitus, but regular biochemical tests should be performed (SoR C). Like with other topical corticoids, there is a risk of developing diabetes mellitus as in humans with the cumulative dose and cumulative duration of use (SoR C) [126].

Neoplastic diseases

No data are available. Hydrocortisone aceponate could be used in dogs with neoplastic diseases after evaluation of the benefit/risk ratio by the responsible veterinarian (SoR C).

Urinary infections

No data are available. Because of its mode of action, hydrocortisone aceponate could be used in dogs with urinary infection (SoR C).

#### 4.2.2. In Atopic Dogs, during the Induction Phase of ASIT

No data are available, but topical glucocorticoids can be used during the induction period of ASIT (SoR C).

#### 4.2.3. In Atopic Dogs, during Allergy Testing

Hydrocortisone aceponate and other topical glucocorticoids should be stopped at least two weeks prior to IDST as they can interfere with the cutaneous reactions (SoR B) [127,128]. No data are available but topical glucocorticoids should not interfere with specific allergen serology tests (SoR C).

#### 4.2.4. In Atopic Dogs, during Dietary Trials

No data are available, but topical glucocorticoids can be used at the beginning of trials for localized lesions to relieve the animal (SoR C).

#### 4.2.5. In Pruritic Ectoparasitic Dermatoses

Topical glucocorticoids can be used when initiating treatment for flea allergy dermatitis (SoR B). With the exception of demodicosis (confirmed or previous), for which topical glucocorticoids should be avoided, a short course of treatment would be acceptable when initiating the treatment of a flea- or sarcoptic-mange-induced pruritus (SoR C).

#### 4.2.6. With Vaccines

No data are available, but because of the absence of a systemic impact, hydrocortisone aceponate can be used (SoR C). Caution is required with other glucocorticoids, depending on their potency, especially when using live attenuated vaccines (SoR C).

### 4.3. Antihistamines

#### 4.3.1. In Atopic Dogs, Excluding the Induction Period of ASIT

##### During Flares (Reactive Therapy)

Antihistamines are not recommended for the treatment of severe acute flare-ups of AD (SoR A). However, because their actions are animal-dependent and often mild, as they are relatively fast acting, mainly inexpensive and have few side effects, they can be used for mild AD flare-ups (SoR C).

##### In Non-Flare Periods (Proactive Therapy)

Limited evidence-based clinical data are available concerning the antipruritic effect of antihistamines and there is a quasi-absence of knowledge concerning the doses and frequency of administration of antihistamines in dogs. Antihistamines could have a global moderate antipruritic efficiency with individual variation (SoR B). Their use for long-term management can be tested alone in the case of mild to moderate dermatosis or associated with other molecules for any degree of severity of dermatosis (SoR C). Indeed, antihistamines can be used in a multimodal approach to reduce the dose of other drugs, such as corticosteroids (SoR C).

##### In Dogs with Superficial Pyoderma or Microbial Overgrowth

Antihistamines can be used in dogs with superficial pyoderma or microbial overgrowth (SoR C).

##### In Dogs with Specific Problems

Liver disorders

No data are available. First-generation antihistamines mainly have a hepatic metabolism. In humans, a dose reduction is recommended in the case of hepatic insufficiency, but their use is not contraindicated [129,130]. So, these molecules could be used in dogs with liver disorders, perhaps at a lower dose (SoR C).

Renal disorders

No data are available. Some antihistamines such as cetirizine have a renal metabolic route in humans, but the route is still not known in dogs [70]. In humans, in the case of renal insufficiency, a reduction in dose is recommended, but its use is not contraindicated [129,130]. Antihistamines could be used in dogs with renal disorders, perhaps at a lower dose (SoR C).

Diabetes mellitus

No data are available. Antihistamines appear to be usable in dogs with diabetes mellitus (SoR C).

Neoplastic diseases

No data are available. Antihistamines appear to be usable in dogs with neoplastic diseases (SoR C).

Urinary infections

No data are available. Antihistamines appear to be usable in dogs with urinary infections (SoR C).

#### 4.3.2. In Atopic Dogs, during the Induction Period of ASIT

Antihistamines can be used during desensitization in dogs (SoR C).

#### 4.3.3. In Atopic Dogs, during Allergologic Tests

A therapeutic window of seven to ten days is necessary before performing an IDST (SoR A). There have been no studies on the impact of antihistamines on serological tests. The mode of action of antihistamines should not interfere with the measurement of allergen-specific IgE. Thus, the withdrawal of antihistamines does not appear to be needed.

#### 4.3.4. In Atopic Dogs, during Dietary Trials

No data are available. Antihistamines are not contraindicated (SoR C) [131].

#### 4.3.5. In Pruritic Ectoparasitic Dermatoses

Antihistamines can be used in cases of mild to moderate parasitic pruritus (SoR C).

#### 4.3.6. During Vaccines

No data are available. The summaries of product characteristics of the available formulations do not limit the use of antihistamines during vaccination. Antihistamines can be used alongside vaccines (SoR C).

### 4.4. Ciclosporin A

#### 4.4.1. In Atopic Dogs, excluding the Induction Period of ASIT

##### During Flares (Reactive Therapy)

Ciclosporin is partially effective at the beginning of treatment, and is fully effective after four to six weeks of treatment (SoR A); it is therefore not recommended for the treatment of flares (SoR A).

##### In Non-Flare Periods (Proactive Therapy)

Ciclosporin is recommended to manage canine AD in non-flare periods (SoR A). It is possible to reduce the frequency of administration after two to four months depending on the clinical response, while maintaining the efficacy of the molecule with treatment on alternate days or twice a week (SoR A). A decrease in the daily dose is also possible (SoR B).

At the beginning of treatment, the combination of ciclosporin (5 mg/kg/day) and prednisolone (1 mg/kg/day for 7 days, then eod for 14 days), accelerates the clinical response with no increase in severe adverse effects (SoR B), and with a threefold greater effect on pruritus in the first few days. The authors suggest modifying the protocol by rapidly reducing the prednisolone dose to 0.5 mg/kg/d (SoR C).

EFA supplementation in the plateau stage (after two months) could improve pruritus and clinical signs (SoR B) and reduce the dose of ciclosporin (from 0.85 mg/kg/day).

Gastrointestinal side effects, most often transient and moderate, are common (46% of cases) (SoR A) [99]. Neoformations are rare (SoR A). Papillomatous lesions are reported, usually not linked to the presence of papillomaviruses, but possibly in response to bacterial infections [132]. These lesions and gingival hyperplasia usually regress after stopping ciclosporin (SoR B).

Infections were present in 11% of treated animals [133]. As the infections were mostly cutaneous (68%), it is difficult to confirm a role for the molecule given the naturally high frequency of infections during CAD and in the absence of studies with control groups [134]. Bacterial urinary tract infections (30% of infections mentioned) are neither statistically more frequent than in untreated dogs, nor are they accompanied by clinical urinary signs [98]. A link between clinical urinary bacterial infection and the use of the drug has therefore not been established, although caution is recommended and regular urinary analyses may be required during long-term treatment (SoR B).

##### In Dogs with Superficial Pyoderma or Microbial Overgrowth

No available information indicates any adverse effects of ciclosporin in dogs with canine AD and superficial infections, and proper management will reduce the frequency of these infections. Ciclosporin is advisable for superficial bacterial infections in dogs with well-managed canine AD (SoR C).

##### In Dogs with Specific Problems

Liver disorders

Ciclosporin, even when used at high doses and for long periods, does not appear to be hepatotoxic in dogs [133], but no data are available in the case of pre-existing liver disorders. The molecule does not seem to be advisable in animals with hepatic disorders because of its mainly hepatic metabolism via cytochrome P-450 [135]. However, ciclosporin seems to be beneficial to hepatocytes during porto-cavity shunting and improves hepatic biochemical parameters during chronic idiopathic hepatitis [136,137].

Ciclosporin is not recommended for liver disorders, but the benefit–risk balance should be assessed (SoR C).

Renal disorders

Unlike in humans, even when used at high doses and for long periods, ciclosporin does not appear to be nephrotoxic in dogs [133] and no adverse effects have been reported in studies on canine AD. No abnormalities in renal biochemical parameters were detected experimentally after two years of treatment in 38 dogs [87]. However, to the best of the authors’ knowledge, no studies have been conducted on animals with renal failure. Ciclosporin seems to be suitable for use in dogs with renal disorders, but the benefit–risk balance should be assessed and regular biochemical tests performed (SoR C).

Diabetes mellitus

Observations of diabetes mellitus in dogs monitored in studies are limited to one case of hyperglycemia and diabetes mellitus after three years of treatment with ciclosporin [12]. Clinical data suggest that treatment in West Highland white terriers is associated with increased incidence of diabetes mellitus [138]. Experimentally, ciclosporin may reversibly increase peripheral insulin resistance and decrease insulin secretion [139]. In a study of 16 dogs with canine AD, ciclosporin (5 mg/kg/day; six weeks) significantly increased glucose levels and decreased serum insulin concentrations, whereas fructosamine levels (statistically increased) remained within the normal range and no dog developed diabetes mellitus [93]. In diabetic dogs, the clearance of ciclosporin increased significantly and the half-life shortened compared with controls [134]. This led to the recommendation to avoid treating diabetic dogs with ciclosporin (SoR C).

Neoplastic diseases

No increase in the prevalence of neoplasia has been reported in published clinical studies. One study of 51 dogs given ciclosporin for six to 30 months did not show increased neoplasia compared with the general population [87], and ciclosporin treatment in canine AD was not a risk factor for cutaneous lymphoma [140]. No articles analyzed the effect of ciclosporin in animals with neoplastic diseases. However, the immune effects and the potential impact on tumor progression led to the recommendation to avoid using ciclosporin in dogs with neoplasia or when neoplasia is diagnosed during treatment (SoR C).

Urinary tract infections

Ciclosporin can be used in dogs with occasional urinary tract infections (SoR B) but is not recommended in dogs with recurrent episodes of bacterial cystitis (SoR C) [121].

#### 4.4.2. In Atopic Dogs, during the Induction Period of ASIT

No data are available. An increase in the population of regulatory T cells could play a role in desensitization [141]. Ciclosporin administration increases populations of regulatory T cells in humans [142], and has no effect in dogs [143], but never reduces this population. Ciclosporin could react in synergy or have no effect on desensitization. Ciclosporin is usable during desensitization in dogs (SoR C).

#### 4.4.3. In Atopic Dogs, during Allergologic Tests

In addition to the sole published article, one other study showed that ciclosporin reduced reactivity to the skin tests, but not the IgE assay, in relation to an *Ascaris* allergen [144], whereas two abstracts, one from an experimental study and the other from a clinical study, reported no effect [145,146].

Ciclosporin can be used without a therapeutic window before these sensitization tests are performed (SoR B).

#### 4.4.4. In Atopic Dogs, during Dietary Trials

Because ciclosporin is too slow-acting to control pruritus during the induction period of a hypoallergenic diet, and because of its persistent action several days after discontinuation, potentially masking the response to the challenge diet, this molecule is not recommended during a dietary trial (SoR C).

#### 4.4.5. In Pruritic Ectoparasitic Dermatoses

External antiparasitics are rapidly effective, whereas ciclosporin is slow, so its use is not recommended (SoR C).

#### 4.4.6. During Vaccines

There are no published effects of ciclosporin on the efficacy and hazards of vaccination. Only indications of the summary of the product characteristics of ciclosporin-containing drugs are available: do not vaccinate with a live vaccine during treatment or in the two weeks preceding or following treatment (SoR C).

### 4.5. Oclacitinib

#### 4.5.1. In Atopic Dogs, excluding the Induction Period of Allergen-Specific Immunotherapy

##### During Flares (Reactive Therapy)

Due to the rapid and efficient reduction in pruritus, oclacitinib (SoR A) is effective for the treatment of flares, with efficacy obtained in a few hours (SoR B) or a few days (SoR A). The dose of oclacitinib is 0.4–0.6 mg/kg twice a day orally for 14 days, thereafter reduced to 0.4–0.6 mg/kg once a day (SoR A). When tapering oclacitinib therapy, a slight rebound of pruritus is observed (SoR B).

When the molecule chosen for the control of canine AD is ciclosporin, oclacitinib can be used concomitantly at the beginning of the treatment to accelerate the reduction in pruritus (SoR B) during the flare.

Hydrocortisone aceponate can also be combined with oclacitinib to prevent the relapse of pruritus and skin lesion when reducing the frequency of administration of oclacitinib from bid to sid (SoR A).

##### In Non-Flare Periods (Proactive Therapy)

Oclacitinib is safe and effective and is recommended for the long-term management of canine AD (SoR A). Adverse events associated with oclacitinib are uncommon (mainly gastro-intestinal) (SoR B).

##### In Dogs with Superficial Pyoderma or Microbial Overgrowth

Oclacitinib could be used when dogs are affected by these infectious diseases, provided the infectious component is concomitantly—or ideally previously—controlled (SoR C). No study has shown that administration of oclacitinib aggravates the clinical signs of bacterial overgrowth, superficial pyoderma or *Malassezia* dermatitis. Moreover, allergic dogs treated with oclacitinib received less antibiotics than allergic dogs treated with other anti-pruritic treatments [106].

##### In Dogs with Specific Problems

Liver disorders

No data are available. In one case of necrolytic migratory erythema syndrome, oclacitinib was effective as long-term therapy (12 weeks) in controlling pruritus [147]. No study reported adverse hepatic effects in atopic dogs treated with oclacitinib. It thus seems that this molecule can be used in dogs with hepatic troubles, but the benefit–risk balance should be evaluated and a biochemical follow-up test conducted regularly (SoR C).

Renal disorders

No study reported adverse renal effects in atopic dogs treated with oclacitinib. It thus appears that this molecule can be used in dogs with renal troubles, but the benefit–risk balance should be evaluated and the biochemical profile checked regularly (SoR C).

Diabetes mellitus

No study reported signs of diabetes mellitus in atopic dogs treated with oclacitinib. It thus seems that this molecule can be used in dogs with diabetes mellitus, but the benefit–risk equation should be evaluated and the biochemical profile checked regularly (SoR C).

Neoplastic diseases

The summary of product characteristics prevents the use of this molecule in dogs with neoplastic diseases. The development of papilloma has also been reported in dogs. However, there is no association between the daily administration of oclacitinib and the odds of malignancies or benign skin masses [104]. Oclacitinib was a therapeutic option in one case of cutaneous epitheliotropic T-cell lymphoma [148]. Oclacitinib was well tolerated when administered in combination with carboplatin or doxorubicin in nine tumoral cases [149]. Oclacitinib was shown to modulate the production of interleukin-8 and monocyte chemoattractant protein-1 by certain canine MCT cell lines [150]. Oclacitinib appears to be able to reduce pruritus in dogs affected with cutaneous tumors (SoR B).

Urinary tract infections

Some authors observed urinary tract infection/cystitis [100,102] but bacteriuria is not an expected adverse effect in dogs with no prior history of urinary tract infection or predisposed condition. Oclacitinib can be used in dogs with occasional urinary tract infections (SoR B), but it is not recommended for dogs with recurrent episodes of bacterial cystitis (SoR C) [121].

#### 4.5.2. In Atopic Dogs, during the Induction Period of ASIT

No studies have assessed the impact of oclacitinib during desensitization. Some dogs were receiving oclacitinib while being desensitized, but the consequences were not assessed (SoR C) [109]. Because of its mechanism of action, oclacitinib seems usable during the induction period of ASIT with no negative impacts (SoR C).

#### 4.5.3. In Atopic Dogs, during Allergy Testing

No studies have assessed the impact of oclacitinib during allergy testing in atopic dogs. One pilot study used experimental models of canine AD and showed no influence of oclacitinib on allergen-specific IgE results. Oclacitinib can be used during allergy testing (SoR C).

#### 4.5.4. In Atopic Dogs, during Dietary Trials

No negative effect on dietary trials has been published and oclacitinib can be used (SoR C).

#### 4.5.5. In Pruritic Ectoparasitic Dermatoses

With the exception of demodicosis (confirmed or previous), in which case oclacitinib should be avoided (SoR C), a short course of treatment would be acceptable when initiating the treatment of a highly pruritic sarcoptic mange or flea infestation (SoR C).

#### 4.5.6. With Vaccines

No data are available. In the summary of product characteristics, oclacitinib administration was associated with an adequate immune response to modified live vaccine canine distemper virus and canine parvovirus vaccination, but a decreased serological response to modified live vaccine canine parainfluenza and inactivated rabies vaccine. Oclacitinib can be used depending on the vaccine type (SoR C).

### 4.6. Lokivetmab

#### 4.6.1. In Atopic Dogs, excluding the Induction Period of ASIT

##### During Flares (Reactive Therapy)

Lokivetmab subcutaneous injection results in an impressive improvement in pruritus between 4 and 24 h (SoR A) and is effective for the treatment of flares of canine AD (SoR A). Reductions in the pruritus score and in the lesional score were significant 28 days after one injection (SoR B). The lokivetmab dose used in Europe is a minimum of 1 mg/kg, and 2 mg/kg in North America.

##### In Non-Flare Periods (Proactive Therapy)

Lokivetmab is recommended for non-flare periods of canine AD (SoR A). The lesional score improved after the first injection and continued to improve after the second and the third injections (SoR C). Lokivetmab can delay disease flares in some atopic dogs (SoR C).

##### In Dogs with Superficial Pyoderma or Microbial Overgrowth

Lokivetmab can be recommended in dogs with canine AD and superficial pyoderma or microbial overgrowth (SoR C), even if, to date, no data are available to assess the efficacy of lokivetmab or the impact of lokivetmab on dogs with superficial pyoderma or microbial overgrowth. No data are available that suggest a pro-infectious role of lokivetmab when used in dogs suffering from allergic dermatitis (SoR C).

##### In Dogs with Specific Problems

Liver disorders

No specific study has been performed, but lokivetmab does not cause changes in biochemical parameters (SoR C). Lokivetmab can thus be recommended in dogs with liver disorder (SoR C).

Renal disorders

No data are available. Renal parameters are not modified by lokivetmab (SoR C). Lokivetmab thus seems to be usable in dogs with renal disorder (SoR C).

Diabetes mellitus

No data are available. Lokivetmab does not stimulate gluconeogenesis (SoR C). Lokivetmab thus seems to be usable in dogs with diabetes mellitus (SoR C).

Neoplastic diseases

Few data are available. A monthly injection of lokivetmab at a dose of 2 mg/kg was effective in resolving and maintaining pruritus remission over a period of 15 months in a dog with widespread cutaneous mastocytosis [151] (SoR C). Similarly, it was used at a dose of 1.25 mg/kg in a dog with cutaneous epitheliotropic lymphoma in combination with prednisolone and resolved pruritus [152] (SoR C). Lokivetmab can be used in dogs with mastocytosis or epitheliotropic lymphoma and for other neoplastic diseases; however, although its use seems to be safe, there are no data on the safety of lokivetmab in the presence of tumor (SoR C).

Urinary infections

In clinical trials, lokivetmab does not induce urinary infections, or changes in biochemical, hematological, and renal parameters (SoR C). Lokivetmab can thus be recommended in dogs with urinary infections (SoR C).

#### 4.6.2. In Atopic Dogs, during the Induction Period of ASIT

No negative effect on ASIT has been published and lokivetmab can be used during the induction period of ASIT (SoR C).

#### 4.6.3. In Atopic Dogs, during Allergy Testing

No published data are available. Lokivetmab could be used during allergy tests without the need for the withdrawal of the drug before the tests are performed (SoR C).

#### 4.6.4. In Atopic Dogs, during Dietary Trials

No negative effect on dietary trials has been reported and lokivetmab can be used (SoR C). However, due to its prolonged effect beyond six weeks in some dogs, the interpretation of the outcome of the eviction regime should be made with caution.

#### 4.6.5. In Pruritic Ectoparasitic Dermatoses

No data are available. A single injection would be acceptable when initiating the treatment of a highly pruritic sarcoptic mange or FBH (SoR C).

#### 4.6.6. With Vaccines

No data are available. The summary of product characteristics does not limit the use of lokivetmab during vaccination, except to recommend giving the injections at different time points. Lokivetmab can be used with vaccines (SoR C).

## 5. Discussion and Conclusions

The aim of this extensive review was not to rank the molecules for each specific clinical situation, but to provide clinicians with the information they need to make a choice, including information on the presence or absence of data on efficacy, as well as on adverse effects.

Moreover, guidelines should always be interpreted as general recommendations that are appropriate for a large majority of cases based on good clinical practice. The guidelines are established to improve the quality and safety of care but are not intended to describe the entire management of a disease, and cannot cover all clinical situations encountered in routine practice. They do not exempt the clinician from exercising discernment in their management of the patient. These guidelines should be considered as a basis for decision making with regard to which different or additional approaches may be used in certain cases and according to the owner’s preferences. Additionally, it should be borne in mind that the owners have to bear the cost of treatment, especially in the case of chronic diseases such as atopic dermatitis.

In published papers, the number of dogs that did not respond to the antipruritic molecule evaluated is rarely given, which may lead to an overestimation of the efficacy of the molecule. Precise data on adverse effects are often missing and causality is often difficult to establish.

Finally, the use of these molecules must take into account the conditions of use (age, weight, and physiological state) and the relative risks due to drug interactions.

## Figures and Tables

**Table 1:** Level of evidence (LoE) in an individual study of the use of systemic glucocorticoids (dexamethasone, methylprednisolone, and prednisolone) in different clinical situations.

Clinical Situation	Molecule	LoE1	LoE2	LoE3
In atopic dogs, excluding the induction period of allergen-specific immunotherapy	dexamethasone	-	[10]	-
	methylprednisolone	[11,12,13]	[14,15,16,17,18]	[19]
	prednisolone	[20,21,22,23,24,25,26]	[18,27,28,29,30,31,32,33,34,35]	-
In atopic dogs, during the induction period of allergen-specific immunotherapy	methylprednisolone	-	[36]	-
	prednisolone	-	[37,38]	-
In atopic dogs, during allergologic tests	prednisolone	-	[39,40]	-
In atopic dogs, during dietary trials	prednisolone	-	[41,42]	-
In pruritic ectoparasitic dermatoses	-	-	-	-

**Table 2:** Summary of adverse reactions reported in the literature with antipruritic drugs used in dogs.

Antipruritic Drug	Reported Adverse Reactions
systemic glucocorticoids	polyphagia, polyuria–polydipsia, digestive signs, superficial pyoderma
topical glucocorticoids	skin atrophy, polyphagia, polyuria–polydipsia, digestive signs
antihistamines	sedation
ciclosporin	digestive signs, anorexia, infectious skin complications, polyphagia, hypersalivation, abdominal pain, weight loss, gingival hyperplasia, papillomatous lesions, hypertrichosis, lethargy, weakness, pruritus, neurological signs
oclacitinib	digestive signs, otitis, pyoderma, pododermatitis, urinary tract infection
lokivetmab	-

**Table 3:** Level of evidence (LoE) in individual studies of the use of topical glucocorticoids in different clinical situations.

Clinical Situation	Molecule	LoE1	LoE2	LoE3
In atopic dogs, excluding the induction period of allergen-specific immunotherapy	hydrocortisone aceponate	[43,44,45]	[46,47]	[48]
	triamcinolone acetonide	[49]	-	-
In atopic dogs, during the induction period of ASIT		-	-	-
In atopic dogs, during allergologic tests	hydrocortisone aceponate	-	-	[50]
In atopic dogs, during dietary trials		-	-	-
In pruritic ectoparasitic dermatoses	hydrocortisone aceponate	-	[51]	-

**Table 4:** Level of evidence (LoE) of individual studies of the use of antihistamines in different clinical situations.

Clinical Situation	Molecule	LoE1	LoE2	LoE3
In atopic dogs, excluding the induction period of allergen-specific immunotherapy	AHR-13268	-	[52]	-
	cetirizine	[53]	[54,55]	[56]
	clemastine	-	[18,57,58,59,60,61]	-
	cyproheptadine	-	[57,58,62]	-
	chlorpheniramine	-	[57,58,61]	-
	dimetinden	[63]	-	-
	diphenhydramine	-	[61]	-
	fexofenadine	-	[17]	[19]
	hydroxyzine	-	[57,58,61]	[56]
	oxatomide	-	[64]	-
	pheniramine	-	-	[34]
	promethazine	-	[57]	-
	terfenadine	-	[65]	[66,67,68]
	trimeprazine	-	[57]	-
	Chlorpheniramine + hydroxyzine	[63]	[69]	-
In atopic dogs, during the induction period of ASIT	-	-	-	-
In atopic dogs, during allergologic tests	cetirizine	-	-	[40,70,71,72,73]
	diphenhydramine	-	-	[72]
	hydroxyzine	-	-	[70]
	loratidine	-	-	[73]
	terfenadine	-	-	[73]
In atopic dogs, during dietary trials	-	-		-
In pruritic ectoparasitic dermatoses	-	-	-	[66,67,68]

**Table 5:** Level of evidence (LoE) of individual studies on the use of systemic ciclosporin in different clinical situations.

Clinical Situation	LoE1	LoE2	LoE3
In atopic dogs, excluding the induction period of allergen-specific immunotherapy	[12,13,20,24,26,47,74,75,76,77,78,79]	[32,33,80,81,82,83,84,85,86,87,88,89,90,91]	[92,93,94,95]
In atopic dogs, during the induction period of ASIT	-	-	-
In atopic dogs, during allergologic tests	[96]	-	
In atopic dogs, during dietary trials	-	-	-
In pruritic ectoparasitic dermatoses	-	-	-

**Table 6:** Level of evidence (LoE) of individual studies of the use of oclacitinib in different clinical situations.

Clinical Situation	LoE1	LoE2	LoE3
In atopic dogs, excluding the induction period of allergen-specific immunotherapy	[23,44,74,100,101]	[32,86,102,103,104,105,106,107,108]	-
In atopic dogs, during the induction period of ASIT	-	[109]	-
In atopic dogs, during allergologic tests		[105]	
In atopic dogs, during dietary trials	-	[42]	-
In pruritic ectoparasitic dermatoses	-	[110]	-

**Table 7:** Level of evidence (LoE) of individual studies on the use of lokivetmab in different clinical situations.

Clinical Situation	LoE1	LoE2	LoE3
In atopic dogs, excluding the induction period of allergen-specific immunotherapy	[75,111,112,113]	[114,115]	[116,117]
In atopic dogs, during the induction period of ASIT	-	[109]	-
In atopic dogs, during allergologic tests	-	-	-
In atopic dogs, during dietary trials	-	-	-
In pruritic ectoparasitic dermatoses	-	-	-

**Table 8:** Summary of antipruritic drugs that may be used, according to the clinical situation.

Clinical Situation		Molecules That Can Be Recommended
Dog with atopic dermatitis	During flares (reactive therapy)	oral glucocorticoids ((SoR A)) ^1^ topical glucocorticoids ((SoR C)) antihistamines (mild AD flares) ((SoR C)) oclacitinib (SoR A) lokivetmab (SoR A)
	In non-flares periods (proactive therapy)	topical glucocorticoids (SoR A) antihistamines (SoR B) ciclosporin (SoR A) oclacitinib (SoR A) lokivetmab (SoR A)
	With superficial pyoderma or microbial overgrowth	Antihistamines (SoR C) ciclosporin (SoR C) oclacitinib (SoR C) lokivetmab (SoR C)
	During the induction period of ASIT	oral glucocorticoids (SoR B) topical glucocorticoids (SoR C) antihistamines (SoR C) oclacitinib (SoR C) lokivetmab (SoR C)
	During allergologic tests	oral glucocorticoids (serological tests only) (SoR B) antihistamines (serological tests only) (SoR A) ciclosporin (SoR B) oclacitinib (SoR C) lokivetmab (SoR C)
	During dietary trials	oral glucocorticoids (SoR C) topical glucocorticoids (SoR C) antihistamines (SoR C) oclacitinib (SoR C)
In pruritic ectoparasitic dermatoses		oral glucocorticoids (short course) (except demodicosis) (SoR C) antihistamines (SoR C) oclacitinib (except demodicosis) (SoR C) lokivetmab (SoR C)

^1^ The strength of recommendation (SoR) was ranked as follows [8]: A—recommendation based on consistent and good-quality patient-oriented evidence; B—recommendation based on inconsistent or limited-quality patient-oriented evidence; C—recommendation based on consensus, opinion, case studies, or disease-oriented evidence.

**Table 9:** Summary of antipruritic drugs that may be used in atopic dogs with co-morbidities.

Type of Co-Morbidity	Antipruritic Drug That Can Be Recommended and SoR ^1^
liver disorder	topical glucocorticoids (mainly hydrocortisone aceponate) (SoR C) antihistamines (SoR C) oclacitinib (SoR C) lokivetmab (SoR C)
renal disorder	topical glucocorticoids (mainly hydrocortisone aceponate) (SoR C) antihistamines (SoR C) ciclosporin (SoR C) oclacitinib (SoR C) lokivetmab (SoR C)
diabetes mellitus	topical glucocorticoids (hydrocortisone aceponate) (SoR C) antihistamines (SoR C) oclacitinib (SoR C) lokivetmab (SoR C)
neoplastic diseases	topical glucocorticoids (hydrocortisone aceponate) (SoR C) antihistamines (SoR C) oclacitinib (SoR B) lokivetmab (SoR C)
urinary infections	oral glucocorticoids (occasional urinary tract infections) (SoR C) topical glucocorticoids (hydrocortisone aceponate) (SoR C) antihistamines (SoR C) ciclosporin (occasional urinary tract infections) (SoR B) oclacitinib (occasional urinary tract infections) (SoR B) lokivetmab (SoR C)

^1^ The strength of recommendation (SoR) was ranked as follows [8]: A—recommendation based on consistent and good-quality patient-oriented evidence; B—recommendation based on inconsistent or limited quality patient-oriented evidence; C—recommendation based on consensus, opinion, case studies, or disease-oriented evidence.

## Data Availability

The data and the references presented in this study are available from the corresponding author upon request.

## Abbreviations

AD	atopic dermatitis
ASIT	allergen-specific immunotherapy treatment
CADESI	Canine Atopic Dermatitis Extent Severity Index
pVAS	pruritus Visual Analog Scale
LoE	level of evidence
SoRT	strength of recommendation taxonomy
SoR	strength of recommendation
sid	once daily (*semil in die*)
bid	twice daily (*bis in die*)
tid	thrice daily (*ter in die*)
eod	every other day
IDST	intradermal skin tests
QoL	quality of life
